# Direct Neuronal Reprogramming for Disease Modeling Studies Using Patient-Derived Neurons: What Have We Learned?

**DOI:** 10.3389/fnins.2017.00530

**Published:** 2017-09-28

**Authors:** Janelle Drouin-Ouellet, Karolina Pircs, Roger A. Barker, Johan Jakobsson, Malin Parmar

**Affiliations:** ^1^Department of Experimental Medical Science, Division of Neurobiology and Lund Stem Cell Center, Wallenberg Neuroscience Center, Lund University, Lund, Sweden; ^2^John van Geest Centre for Brain Repair and Department of Neurology, Department of Clinical Neurosciences and Cambridge Stem Cell Institute, University of Cambridge, Cambridge, United Kingdom

**Keywords:** induced neurons, direct neural reprogramming, disease modeling, neurological disorders, neurodegenerative diseases

## Abstract

Direct neuronal reprogramming, by which a neuron is formed via direct conversion from a somatic cell without going through a pluripotent intermediate stage, allows for the possibility of generating patient-derived neurons. A unique feature of these so-called induced neurons (iNs) is the potential to maintain aging and epigenetic signatures of the donor, which is critical given that many diseases of the CNS are age related. Here, we review the published literature on the work that has been undertaken using iNs to model human brain disorders. Furthermore, as disease-modeling studies using this direct neuronal reprogramming approach are becoming more widely adopted, it is important to assess the criteria that are used to characterize the iNs, especially in relation to the extent to which they are mature adult neurons. In particular: i) what constitutes an iN cell, ii) which stages of conversion offer the earliest/optimal time to assess features that are specific to neurons and/or a disorder and iii) whether generating subtype-specific iNs is critical to the disease-related features that iNs express. Finally, we discuss the range of potential biomedical applications that can be explored using patient-specific models of neurological disorders with iNs, and the challenges that will need to be overcome in order to realize these applications.

## Introduction

Direct reprogramming of a terminally differentiated cell into another cell type was achieved for the first time in 1987 with the conversion of fibroblasts to myoblasts (Davis et al., 1987). Following this, however, it took more than two decades to successfully directly reprogram fibroblasts to neuronal cells using the forced expression of the neuronal transcription factors *Ascl1, Brn2*, and *Myt1l*- a cell product termed induced neuron (iN) (Vierbuchen et al., 2010). Since then, the field of direct neuronal reprogramming has been applied to human cells and been expanding at a fast pace, and studies using patient derived iNs to model neurological disorders have started to appear.

iN cells, in contrast to induced pluripotent stem cells (iPSCs), are the result of direct reprogramming of one type of somatic cell into another without going through a pluripotent intermediate stage. Because of this feature, it was hypothesized that iNs would therefore retain some of the characteristics of the starting cell, especially related to epigenetic status and aging. Two studies have now demonstrated that this is the case—at least to some extent. Using a broad range of human fibroblasts from different age donors, Mertens et al. demonstrated that iNs exhibit an age-dependent regulation of genes associated with aging. They found that there is an age-dependant loss of nucleocytoplasmic compartmentalization in donor fibroblasts which was kept in iNs but restored in iPSCs derived from aged cells. More specifically, they further demonstrated that RanBP17, a receptor that decreases with aging, was also decreased in an age-dependent manner in iN cells—features that were both absent in iPSCs (Mertens et al., 2015a). Using different approaches to assess the age of the cell that relies on the epigenetic DNA methylation age measurements, a method that looks at a number of genomic loci becoming differentially methylated with age to predict the age of the cell (in years) (Horvath, 2013), Huh et al. have also shown that iNs retain the age of the donors at the epigenetic level. Moreover, they show that the aging signature is maintained through their microRNA expression profile and increased oxidative stress levels (Huh et al., 2016).

Neurons are especially affected by aging given that they do not regenerate in most regions of the brain, which could underlie why the majority of neurodegenerative disorders present clinically later in life. As a result, age is a prominent risk factor in many of these diseases including Alzheimer's disease (AD), Parkinson's disease (PD), amyotrophic lateral sclerosis (ALS) and Huntington's disease (HD). Animal models of these disorders can mimic some aspects of these human-specific diseases but most models are toxin- or vector-based, or use mendelian forms of these diseases as their starting point, and do not recapitulate the appearance of disease phenotypes associated with human aging. As such, there is a pressing need for models that faithfully recapitulate both the sporadic and age related aspects of these common chronic neurodegenerative disorders in human cells.

As the number of disease modeling studies using iNs being published are starting to increase, here we review what has been accomplished to date, and provide an outlook of what could be achieved in the future.

## Can patient-derived iNS provide an authentic cellular system to assess disease-related phenotypes?

Patient specific neurons derived from iPSCs have shown a wide array of disease-associated phenotypes. The majority of those studies have studied mendelian forms of neurological disorders but some features could also be observed in sporadic forms of diseases such as schizophrenia, bipolar disorder and ALS (Koch et al., 2011; Burkhardt et al., 2013; Mertens et al., 2015b). To date, at least ten neurological disorders have been modeled using patient-derived iN cells (see Table 1) and multiple disease-associated phenotypes has been observed, although studies looking at disease features in lines from sporadic patients has yet to be reported. However, while some of these features have been uniquely seen in iN cells, other phenotypes can be detected in the starting cell before conversion or in neurons differentiated from patient-derived iPSCs. Given that each cellular system has their own merits and challenges, it will be important to decipher the benefits that iNs have for modeling neurological disorders. In addition to a much shorter and easier reprogramming route, the most important difference known to date between neurons generated from iPSCs or directly from fibroblasts is the age of the cell. It may be, though, that iPSC derived neurons will be best suited for modeling diseases associated with developmental processes whereas iNs will be most useful to study disorders associated with aging.

**Table 1:** Neurological disease modeling in induced neurons.

**Disease**	**Mutations**	**References**	**Target cell type**	**Reprogramming strategy**	**Days post transduction**	**% Conversion efficiency**	**% Purity**	**iN characterization**	**Electrophysiology**	**Disease phenotype**
PD	*PINK1* Q456X	Fiesel et al., 2015	iN	LV.shPTB, bFGF, BDNF, GDNF, NT3, CNTF	12–14	ND	ND	ICC: TUJ1 WB: MAP2, TUJ1	ND	No pS65-Ub accumulation upon mitochondrial damage
	*PINK1* p.G411S HET, p.Q456X HET or HOMO	Puschmann et al., 2017	iN	LV.shPTB bFGF, BDNF, GDNF, NT3, CNTF	12–14	ND	ND	ICC: TUJ1 WB: TUJ1	ND	Reduced pS65-Ub levels in p.G411S over time elevated parkin levels
FTD and parkinsonism	*MAPT* (K298E)	Iovino et al., 2014	iN	LV.ASCL1-BRN2-MYT1L	30–53	ND	ND	ICC: TUJ1	ND	3R and 4R tau isoform expression
PKAN	*PANK2*	Santambrogio et al., 2015	iDAN	LV.ASCL1-NURR1-LMX1a	20	5	ND	ICC: TUJ1, NCAM, MAP2	ND	Altered oxidative status mitochondrial dysfunction
								TH (50% of TUJ1+)		
AD	*APP* (V717I) or *PSEN* (I167 or A434T or S169del)	Hu et al., 2015	ciN or iN	small molecules VCRFSGYD bFGF, cAMP, BDNF, GDNF, NT3 or LV.ASCL1-NGN2 and cAMP, SB, noggin, LDN, CHIR, BDNF, GDNF, NT3	14–28	10–13	ND	ICC: TUJ1, DCX, MAP2, TAU, NEUN, SYN vGLUT	YES	Abnormal Aβ production increased pTau and Tau levels in APP
								RT-qPCR; Single-cell sequencing; microarray; FACS		
HD	*HTT* 68Q or 86Q	Liu et al., 2014	iN	LV.shPTB bFGF, BDNF, GDNF, NT3, CNTF	19–30	ND	8–14	ICC: TUJ1, NEUN (10%), GABA, DARPP32 (60-80%)	ND	Neuritic breakdown, Abnormal neuritic branching, increased cell death aggregation of mutant huntingtin
BD	ND	Bavamian et al., 2015	iN	LV.miR9/9^*^-124 + NEUROD2-ASCL1-MYT1L VPA	38–40	ND	ND	ICC: MAP2, TUJ1	ND	Increased miR-34a levels
Schizophrenia	16p11.2 duplication 22q11.21 deletion	Passeri et al., 2016	iN	LV.ASCL1-BRN2-MYT1L, SB, Noggin, CHIR, cAMP, VPA, BDNF, GDNF, NT3	21	ND	ND	ICC: MAP2	ND	Toxoplasma gondii infection and characterization
	22q11.2 deletion; 16p11.2 duplication and/or 22q13.3 duplication	Passeri et al., 2015	iN	LV.ASCL1-BRN2-MYT1L, SB, noggin, CHIR, cAMP, VPA, UNO, GDNF, BDNF, NT3	21	ND	20–40	ICC: MAP2	ND	Neurons with a similar morphological complexity
	SNPs rs1198588, rs1625579, rs2660304, rs2802535	Siegert et al., 2015	iN	LV.ASCL1-BRN2-MYT1L, bFGF	28	ND	ND	FACS sort (0.01-0.001% iN cells/all FACS events)	ND	Minor allele SNPs cause miR-137 gain of function miR-137 genetic risk
SMA	*SMN1*	Zhang et al., 2017	iMN	LV.ASCL1-NEUROD1-BRN2-MYTL1-NGN2-ISL1-HB9-LHX3 bFGF, BDNF, GDNF, IGF1, cAMP	23–62	6	2–5	ICC: TUJ1, CHAT	ND	Reduced neurite outgrowth, disintegrated neurons, neurodegeneration (day 60), increased caspase-3 levels, high LDH activity
ALS	C9orf72 repeat expansion	Su et al., 2014	iN	LV.shPTB, bFGF, BDNF, GDNF, NT3, CNTF	15–19	ND	ND	ICC: MAP2, TUJ1, SYN, PSD95, SMI32, Drebrin	ND	Cytoplasmic poly(GP) inclusions
	*FUS* p.G504Wfs^*^12 p.R495^*^ or p.Q519e	Lim et al., 2016b	iN	LV.shPTB, bFGF, BDNF, GDNF, CNTF, NT3	10–21	80–90	ND	ICC: TUJ1, MAP2, NEUN, SYN	ND	Reduced endogenous FUS levels in nucleus, increased cytoplasmic FUS levelsneuropathology of FUS mutations with a disrupted NLS region
	*FUS* R522R or H517Q or R521G	Liu et al., 2016	iMN	LV.NGN2-SOX11-ISL1-LHX3 FSK, DM, bFGF, BDNF, GDNF, NT3	14–49	80–93	95–97	ICC: TUJ1, MAP2, NF200, SYT1, HB9, CHAT, VACHT RT-qPCR	YES	Mislocalization of FUS, shrunken somas, deficits in AP firing and reduced membrane capacitance, impaired control of muscle contraction
Krabbe-disease	*GALC* (p.K563^*^;L634S) or (p.N228_S232delinsT; G286D)	Lim et al., 2016a	iN	LV.shPTB bFGF, BDNF, GDNF, CNTF, NT3	8–10	ND	ND	ICC: MAP2, TUJ1, SYN, vGLUT, Phalloidin, TAU WB: TUJ1	ND	Diminished GALC activity, increased psychosine levels, neurite fragmentation, abnormal neuritic branching, higher LAMP1 level, enlarged and fragmented LAMP1+ vesicles, mitochondrial morphology altered

### Disease-associated features unique to human cells

Mouse fibroblasts, especially at the embryonic stage, are easier to reprogram than human adult fibroblasts and the resulting cells mature faster. For example, spontaneous action potentials can be detected in iNs originating from MEFs as early as 8 days into conversion (Vierbuchen et al., 2010), whereas the earliest time point when spontaneous action potentials could be detected to date in human iNs is 46 days (Xu et al., 2015), suggesting that human cells take longer to become fully mature. Therefore, mouse embryonic fibroblasts have been used to study the disease mechanisms in iNs in monogenic disorders (Chanda et al., 2013). While this approach may be a starting point through which to study the impact of a specific mutation on disease pathogenesis, it has been reported that some disease-related phenotypes only have a pathology in human iNs. For example, iNs derived from ALS patients carrying a mutation in the fused in sarcoma (FUS) protein recapitulated the localization of the mutated protein in the cytoplasm instead of the nucleus following stress induction, a feature that rat primary neurons carrying the same mutation failed to express (Lim et al., 2016b). In another study investigating iron metabolism in neurodegeneration with brain iron accumulation (NBIA), mitochondrial iron and energetic dysfunction were observed in both pantothenate kinase-associated (PKAN) patient derived fibroblasts and iNs (Santambrogio et al., 2015), whereas these features were not seen in fly or mouse models of the disease (Rana et al., 2010; Brunetti et al., 2012). While there are only a limited number of such reports published to date, they do highlight some species-specific differences and favor the use of human-based cellular system(s) in which the disease-associated phenotypes will be assessed, especially given these diseases are all uniquely human.

### Disease-associated features present in iNs and absent in parental fibroblasts

Disease-associated features are not always unique to the neurons and as a result, several of these phenotypes can be observed in both iNs and fibroblasts (Santambrogio et al., 2015; Lim et al., 2016a). However, as iNs adopt a neuronal-like morphology and at least some functional properties of neurons, they provide an opportunity to study diseases in the cell type primarily clinically affected. For example, iNs derived from adult-onset Krabbe disease had the same lysosomal storage defects as the starting fibroblasts, but unique to the iNs was the abnormal neuronal branching, which may be more relevant to the clinical expression of this disorder (Lim et al., 2016a). In fact, a few studies have now reported that disease-associated features could only be seen in iNs. For example, Lim et al. (2016b) reported that mutant FUS-associated pathology was observed in iNs derived from familial ALS patients, but not transfected cells or patient-derived fibroblasts (Lim et al., 2016b). In line with this, an independent study in induced motor neurons (iMNs) also reported such disease-associated phenotype (Liu et al., 2016). In another report, *Toxoplasma gondii* infection of iN cells derived from patients with childhood onset schizophrenia resulted in cyst formation due to *T. gondii* differentiation in the iN soma, whereas the infected parental fibroblasts were completely lysed by parasite infection (Passeri et al., 2016). Given that the conversion of the fibroblasts to iN cells allowed the formation of cysts, this argues in favor of the specific need of the relevant neuronal cell type to assess disease pathogenesis. Other examples illustrating this point include a study showing that changes at the level of pathological protein expression have also been observed, with elevated levels of Aβ42 as well as phosphorylated Tau in iNs derived from patients with familial AD as compared to fibroblasts (Hu et al., 2015). Finally, the investigation of phenotypes associated with the repeat expension r(GGGGCC)_exp_ in *C9orf72* that leads to frontotemporal dementia (FTD) and ALS resulted in the detection of cytoplasmic poly(GP) as well as poly(PR) inclusions in iNs but not in fibroblasts (Su et al., 2014).

**Figure 1:**
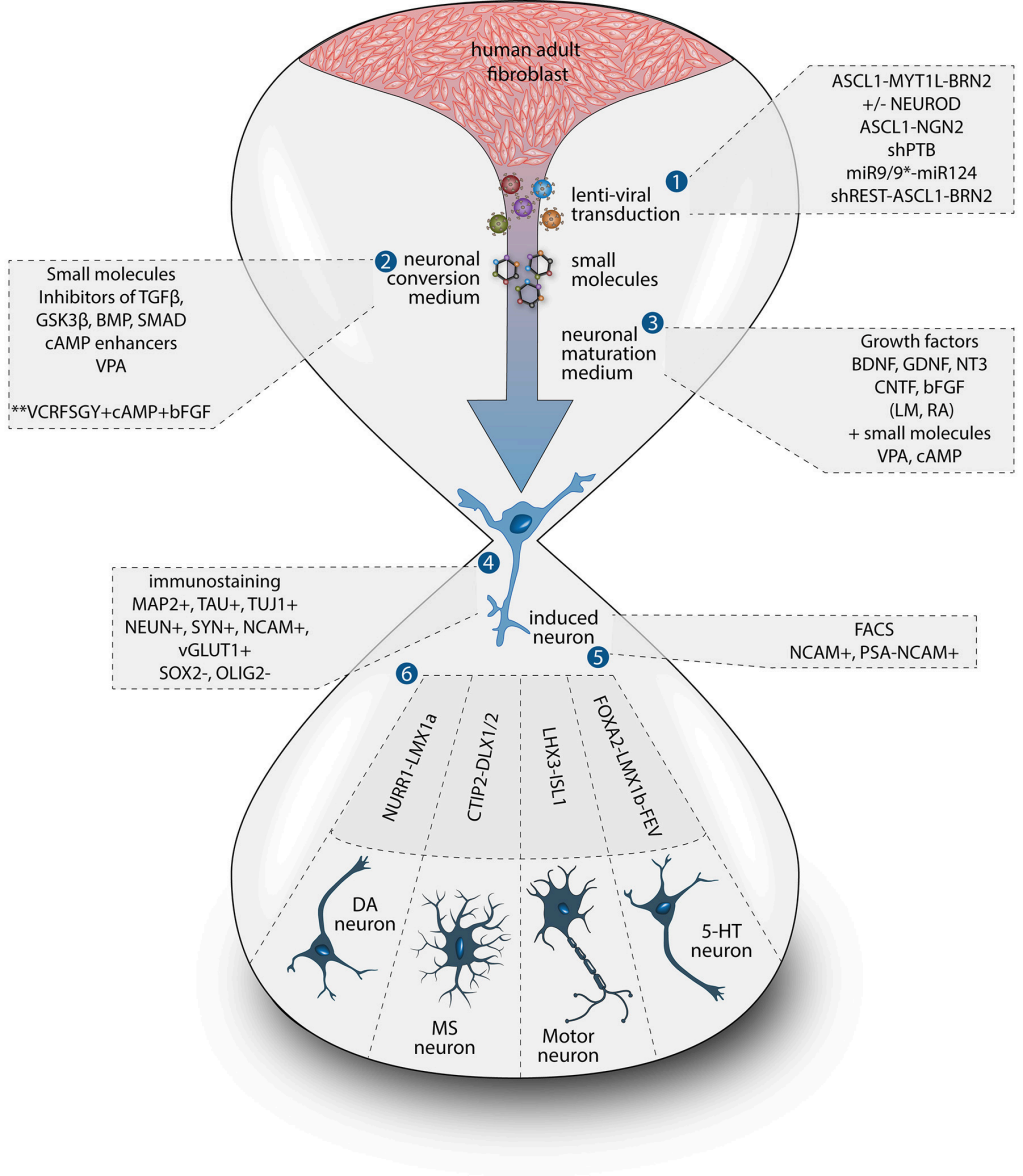
Methods for direct neuronal conversion. During direct neuronal conversion, adult human fibroblasts undergo progressive conversion into iNs. This process is initiated by a lentiviral transduction (1) to deliver the reprogramming factors and/or the addition of chemical compounds (2, 3). Neuronal identity can be confirmed by the expression of pan-neuronal markers (4). iN cultures can further be purified using antibiotic selection and/or cell sorting (5). Subtype specific neurons can also be obtained by the addition of fate determinant reprogramming factors (6).

One important caveat with most of these studies, however, is that the iNs that have been used for disease modeling were mostly at early stages of conversion, and thus rather immature neurons in terms of function, marker expression and morphology. Additionally, most studies have been performed on a pan-neuronal or unspecified neuronal subtype rather than on a specific subtype of neuron. When using iPSCs for disease modeling, subtype specific disease-related features have been reported to be important. For instance, abnormal neuronal arborization was observed in dopaminergic neurons bearing a *Leucine-rich repeat kinase 2 (LRRK2)* mutation but not in sensory neurons differentiated from the same cell source (Schwab and Ebert, 2015). It is thus likely that iNs of different subtypes may express distinct disease-related phenotypes, which will be important to study given that most of these diseases have pathology that is region specific in the CNS/PNS. This ability to generate subtype specific neurons has now been achieved for many types of neurons, including dopaminergic (Caiazzo et al., 2011; Pfisterer et al., 2011a), striatal medium spiny (Victor et al., 2014), cholinergic (Liu et al., 2013; Zhang et al., 2017), nociceptive (Wainger et al., 2015), spinal motor (Son et al., 2011; Liu et al., 2016), GABAergic interneurons and serotoninergic neurons (Xu et al., 2015; Vadodaria et al., 2016) (see Figure 1). Furthermore, the generation of such subtype specific iNs provides the advantage to expand the array of functional assays that can be performed. For example, the formation of functional neuromuscular junctions by iMN could be evaluated in co-cultures with primary mouse skeletal myotubes, as well as through more conventional approaches such as electrophysiologically, a functional aspect which has been shown to be impaired in iMNs derived from ALS patients (Liu et al., 2016).

Ideally, investigation of disease-associated phenotypes should be done using neuron specific subtypes and comparing subtypes of cells that are, or are not, affected in the disease process. For this purpose, the generation of additional subtypes of neurons as well as the optimization of current reprogramming protocols to produce iNs that mimic more closely the cellular phenotypes of the diverse human neuronal subtypes is needed. This, however, will remain challenging as long as the mechanisms behind fate specification during direct reprogramming are not better understood. Furthermore the production of a high yield of subtypes specific iNs is technically very challenging given that this often requires the delivery of a greater number of reprogramming factors and as a result, only a small subset of cells expresses the full set.

## How to define an iN?

The term iN has been used to describe neurons generated from multiple cell sources and through multiple methods. A first important distinction should be made between neurons differentiated from iPSCs using extrinsic factors, neurons obtained from pluripotent stem cells through the forced expression of programming factors or via the direct reprogramming of somatic cells to neuronal progenitors and further differentiated into mature neurons. Here, we define iNs as the product of directly reprogrammed neurons starting from somatic cells, such as a fibroblast, and avoiding a pluripotent or progenitor stage intermediate and we focus on iNs produced from adult human fibroblasts due to their utility in disease modeling.

These type of adult iNs have been generated using four main methods: (i) by the forced expression of transcription factors (Caiazzo et al., 2011; Pfisterer et al., 2011b; Iovino et al., 2014; Mertens et al., 2015a; Passeri et al., 2015; Siegert et al., 2015; Liu et al., 2016), (ii) by knocking down of the RNA-binding proteins PTB/nPTB (Xue et al., 2016) or p16-p19 (Sun et al., 2014), (iii) by the forced expression of neuronal specific microRNAs (Victor et al., 2014; Richner et al., 2015; Huh et al., 2016), (iv) by chemically manipulating pathways involved in neuronal fate and functions (Hu et al., 2015) or by a different combination of these strategies (Ambasudhan et al., 2011; Liu et al., 2013; Hsu et al., 2014; Wang et al., 2014; Xu et al., 2015; Drouin-Ouellet et al., 2017) (Figure 1). Each of these methods has been proven effective in generating functional neurons in which it is possible to evoke action potentials as well as observe spontaneous synaptic activity within a timeframe ranging from 4 to 12 weeks when co-cultured with astrocytes or primary cortical neurons or after transplantation (Hu et al., 2015; Huh et al., 2016; Liu et al., 2016; Xue et al., 2016; Drouin-Ouellet et al., 2017), and even spontaneous action potentials in some cases (Mertens et al., 2015a). The resulting iNs have also been shown to express mature neuronal markers including MAP2, TAU, and NEUN with complex neuronal morphology.

**Figure 2:**
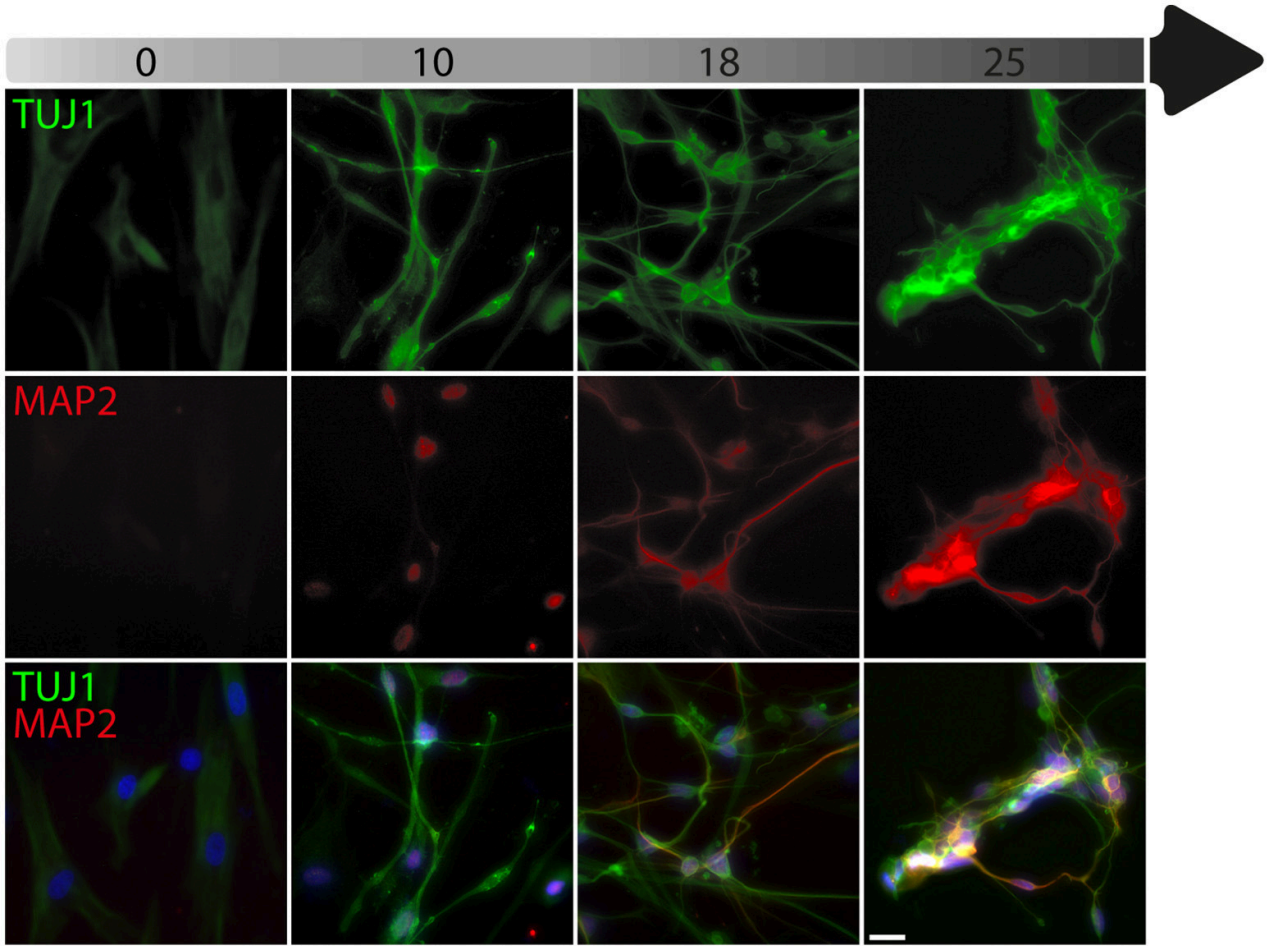
Timeline of neuronal marker expression during reprogramming. Representative images of TUJ1 and MAP2 double immunostaining counterstained with DAPI (in blue) showing low levels of TUJ1 in dermal fibroblasts (in green), followed by intensification of expression at day 10, which is sustained until day 25 post-transduction with the U6.shREST.PGK.BRN2.PGK.ASCL1.WPRE construct. MAP2 expression (in red) is detectable in the nucleus at day 10 and is incrementally expressed in the processes from day 18 to 25. Scale bar = 25 μm.

**Figure 3:**
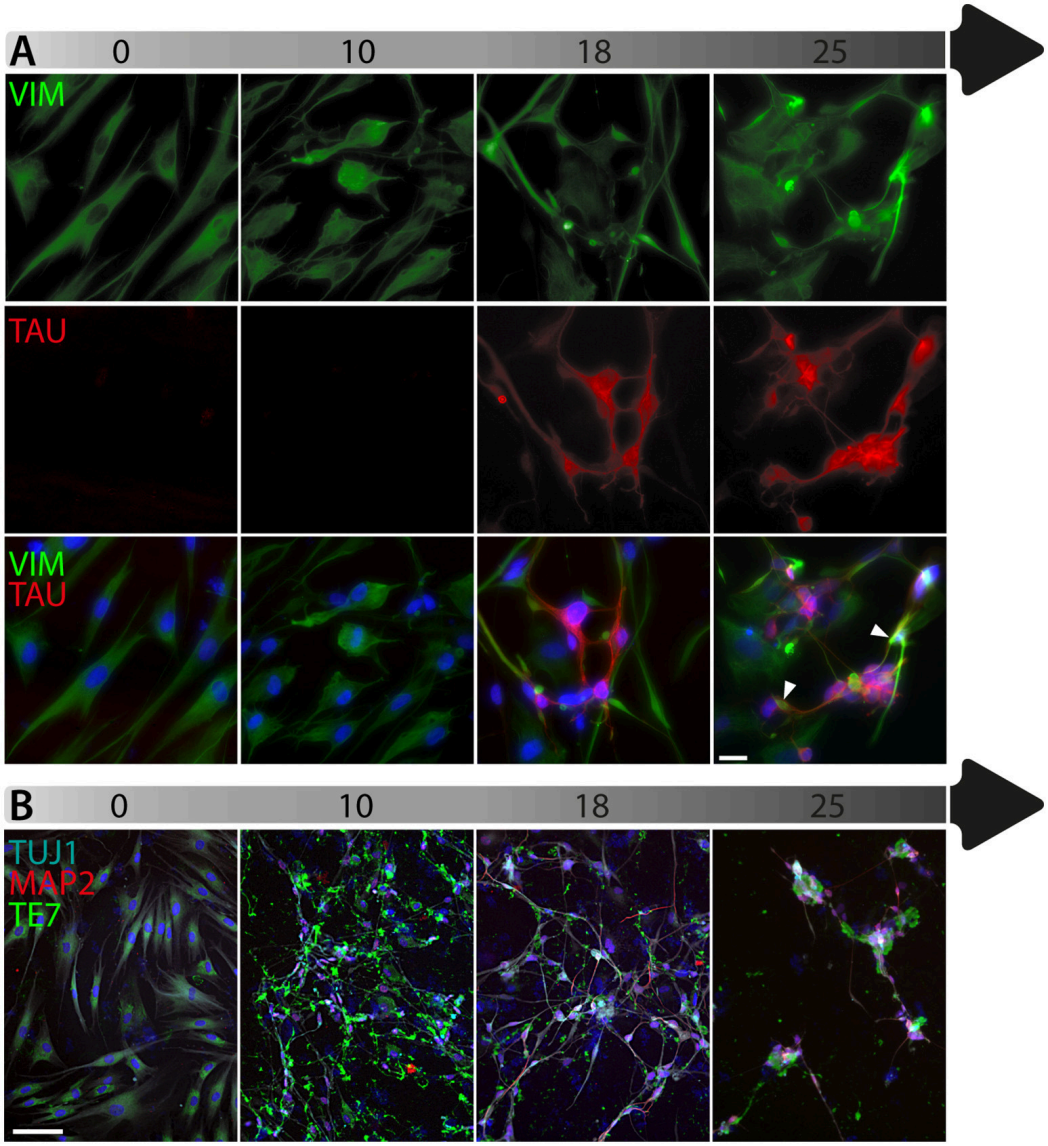
Timeline of fibroblast marker expression during reprogramming. **(A)** Double immunofluorescent staining of the fibroblast and neuronal progenitor marker Vimentin (VIM; in green) and the neuronal marker TAU (in red) counterstained with DAPI (in blue) showing Vimentin expression in dermal fibroblasts as well as in a subpopulation of cells that are not expressing TAU at day 18 and 25 post-transduction. The majority of TAU expressing cells do not express VIM except for a few cells, which are double TAU/VIM+ (white arrowheads). **(B)** TUJ1 (cyan), MAP2 (red) and TE7 (green) triple immunostaining counterstained with DAPI (in blue) showing expression of the fibroblast marker TE7 in fibroblasts before transduction, whereas TUJ1+ and MAP2+ cells are negative for TE7, which is only detectable extracellularly at later time points during conversion. Scale bar in A = 25 μm, B = 100 μm.

To date, the predominant method by which to isolate/identify the iN population to assess disease relevant phenotypes in patient derived iNs, has involved either an antibiotic selection to remove cells that are not expressing the reprogramming construct(s) (Liu et al., 2014; Su et al., 2014; Bavamian et al., 2015; Lim et al., 2016a) or based on the expression of neuronal markers such as TUJ1 (βIII-Tubulin) (Iovino et al., 2014; Liu et al., 2014; Fiesel et al., 2015; Lim et al., 2016a,b; Puschmann et al., 2017). However, direct neuronal reprogramming studies that have used antibiotic selection to purify the neuronal culture have consistently reported that a significant percentage of cells do not convert even though the reprogramming constructs are expressed (Victor et al., 2014; Mertens et al., 2015a; Huh et al., 2016; Liu et al., 2016; Xue et al., 2016), which presents the need to identify the iN population even after antibiotic selection. Furthermore, using a method that combines the forced expression of Ascl1 and Brn2 with the knockdown of the neuronal repressor complex REST, we show that although an important proportion of cells expressing stronger levels of TUJ1 can be observed very early on, only a few MAP2+ or TAU+ cells are detectable around day 18 and this number increases at day 25 (Figure 2). Notably, a striking and progressive change in cell morphology toward a more mature neuronal appearance is observed over time—e.g., a decrease of the size of the nucleus and cell body, thinning and elongation of the processes and increase in the number of branches. As expected, some markers of fibroblasts and non-mature neurons such as Vimentin can be co-expressed with mature neuronal markers in iNs even at later time points (Figure 3A), whereas the marker TE7, which is fibroblast-specific, is not co-expressed in TUJ1+ cells as early as day 10 (Figure 3B). Indeed Xue et al. (2016) have reported that TUJ1 is expressed as early as 3 h following shPTB and plateaus at 1 day post transduction - at a time when a fibroblast marker such as fibronectin is still strongly expressed and when the transduced cells do not exhibit a full neuronal morphology (Xue et al., 2016). These authors have also shown that knocking down PTB is not sufficient to induce the expression of mature neuronal markers such as MAP2 and NEUN in adult human fibroblasts but that the full maturation of iNs requires sequential nPTB knockdown. As a result, the neuronal identity of iNs used in disease modeling studies that have both knocked down PTB and used TUJ1 as a neuronal marker or only antibiotic selection to obtain the iN population, has not been confirmed.

These challenges with iN reprogramming can lead to potential bias when assessing neuron-specific phenotypes associated with different disorders as cells not fully converted or not fully mature may not be an ideal system for modeling of diseases in which the primary cell population affected are neurons. As such, we suggest that disease modeling studies should use:

1. One of the methods that have been proven to generate mature neurons in adult human fibroblasts (Hu et al., 2015; Mertens et al., 2015a; Richner et al., 2015; Xue et al., 2016; Drouin-Ouellet et al., 2017), or new methods where the neuronal identity and function is well documented.
2. The expression of at least one of the following markers (MAP2, TAU, and/or NEUN) to identify the neurons and
3. Morphological criteria for neuronal identification and
4. Conversion protocols with maturation times *in vitro* of at least 4 weeks.

## Future outlook

Although the field of modeling neurological disorders with iN cells is still in its early stages, the results reported thus far support the need for further development of the iN technology, as it has already been shown to be useful in studying some neuronal specific age related human diseases. Up until very recently, the methods used to obtain iNs have been rather inefficient when applied to adult human fibroblasts, which has greatly hampered their utility for disease modeling. To circumvent this issue, we have developed a simple single-step and single-vector based approach that can generate very high yields of iNs from patients with neurodegenerative disorders independent of the passage number of the fibroblasts (Drouin-Ouellet et al., 2017). This new approach results in cells that fulfill the above criteria for iN cell suitable for disease modeling as outlined above (expression of mature neuronal markers, neuronal morphology, more than 4 weeks maturation *in vitro*) and overtime the cells develop functional properties of neurons including post-synaptic currents and the ability to fire action potentials. The simplicity and high efficiency of the method should facilitate the application of direct neuronal reprogramming for disease modeling studies.

The use of iNs is advantageous in terms of allowing studies of large cohort of patients and controls including patients with sporadic diseases within shorter time, with relatively little work and cost compared to iPSC-based modeling. Another advantage of iN cells is that they maintain, at least partially, the aging signature of the cell, and are therefore more likely to provide insights into the molecular mechanisms underlying the age-dependent pathogenesis of some neurological disorders, as well as the pathological basis of their clinical heterogeneity.

Another potential advantage of iNs is that they do not rely on clonal selection and while this could result in a higher heterogeneity of the final neuronal population, the end product as a whole is more likely to be biologically relevant than a few selected iPSC clones. Furthermore, as methods for producing glial cells by direct reprogramming are emerging (Caiazzo et al., 2015; Tian et al., 2016), we can expect a greater sophistication of the induced cellular systems and with this, a more comprehensive assessment of specific non-cell autonomous interactions involving multiple neural cell types during disease processes on a patient-specific basis.

Once models for a diverse range of neurological disorders using iNs have been well established, we anticipate that there will be an expansion of the field toward early and differential diagnostics, drug target validation as well as drug screening assays. However, for this to become a reality, a number of challenges need to be overcome. For instance, careful characterization of the cell product should be carried out in terms of neuronal phenotypes as well as subtype authenticity to mimic as close as possible the types of neurons that are found and affected in the human brain. In that respect, more molecular studies at the single cell level are warranted to better understand the relationship between the reprogramming and the endogenous factors, as well as the target level of expression needed to perfect the end cell product. In support of this, novel reprogramming strategies which ensure that the full set of factors are expressed in each starting cell, and which provide a better control of their expression level will improve the yield of the target iN subpopulations. Finally, further insights into the mechanisms of direct reprogramming will undoubtedly help shed light on how best to bring iN technology to the point where it becomes a routine tool, as well as possible therapeutic approach in its own right.

## Methods

An adult dermal fibroblast line derived from a skin biopsy from a neurologically healthy 71-year-old male was obtained from the Parkinson's Disease Research clinic at the John van Geest Centre for Brain Repair (Cambridge, UK) and used under local ethical approval (REC 09/H0311/88). Written informed consent was obtained from the participant, and the experiments conformed to the principles set out in the WMA Declaration of Helsinki and the Department of Health and Human Services Belmont Report. For details on the skin biopsy sampling and the fibroblast cultures, refer to Drouin-Ouellet et al. (2017).

Neuronal reprogramming was done as described before (Drouin-Ouellet et al., 2017) using a single third-generation lentiviral vector expressing a combination of Ascl1 and Brn2 with short hairpin RNA (shRNA) targeting REST. It was generated with a non-regulated ubiquitous phosphoglycerate kinase (PGK) promoter produced as previously described (Zufferey et al., 1997) and titrated by quantitative PCR (qPCR) analysis (Georgievska et al., 2004). Transduction was performed at a multiplicity of infection (MOI) of 20. The virus titer was 1.93E + 09.

Immunocytochemistry was performed at day 0, 10, 18 and 25 as previously described (Drouin-Ouellet et al., 2017). The following primary antibodies were used in the blocking solution overnight at 4°C: chicken anti-MAP2 (1:15,000; Abcam, ab5392); mouse anti-TUJ1 (1:1,000; Promega, G7121); rabbit anti-TUJ1 (1:1,000; BioLegend, 801201); chicken anti-VIM (1:5,000; Millipore, AB5733); mouse anti-TAU clone HT7 (1:500, Thermo Scientific, MN1000); mouse anti-TE7 (1:100, Millipore, CBL271). On the second day, after washing twice with PBS, Cyanine-conjugated secondary antibodies (1:200; Jackson ImmunoResearch Laboratories) were added and counterstained with DAPI (1:1,000; Sigma-Aldrich). Images were captured from a PBS-filled well at 20X using an inverted microscope (Leica, DFC360 FX-DMI 6000B).

## Author contributions

All authors listed have made a substantial, direct and intellectual contribution to the work, and approved it for publication.

### Conflict of interest statement

The authors declare that the research was conducted in the absence of any commercial or financial relationships that could be construed as a potential conflict of interest. The reviewer AC and handling Editor declared their shared affiliation.

## Abbreviations

5-HT: 5-hydroxytryptamine or serotonin
AD: Alzheimer's disease
ALS: amyotrophic lateral sclerosis
AP: action potential
APP: amyloid precursor protein
ASCL1: Achaete-scute homolog 1
BD: bipolar disease
BDNF: brain-derived neurotrophic factor
bFGF: basic fibroblast growth factor
BMP: bone morphogenetic protein
BRN2 or POU3F2: POU domain, class 3, transcription factor 2
C or CHIR: CHIR99021
cAMP: cyclic adenosine monophosphate
CHAT: choline acetyltransferase
ciN: chemical induced neuron
CNTF: ciliary neurotrophic factor
CNS: central nervous system
CTIP2 or BCL11B: B-Cell CLL/Lymphoma 11B
D or DM: dorsomorphin
DARPP32: dopamine- and cAMP-regulated neuronal phosphoprotein
DCX: doublecortin
DLX1/2: distal-less homeobox 1/2
F: forskolin
FACS: fluorescence-activated cell sorting
FEV: ETS Transcription Factor
FOXA2: forkhead box protein A2
FTD: frontotemporal dementia
FUS: fused in sarcoma
G: GO6983
GALC: galactosylceramidase
GDNF: glial cell-derived neurotrophic factor
GSK3β: glycogen synthase kinase 3 beta
HB9: homeobox gene 9
HD: Huntington's disease
HET: heterozygous
HOMO: homozygous
ICC: immunocytochemistry
iDAN: induced dopaminergic neuron
IGF1: insulin-like growth factor 1
iMN: induced motor neuron
iN: induced neuron
iPSc: induced pluripotent stem cell
ISL1: insulin gene enhancer
LAMP1: lysosomal-associated membrane protein 1
LDH: lactate dehydrogenase
LHX3: LIM/homeobox
LM: LM-22A4
LMX1a/b: LIM homeobox transcription factor 1, alpha or beta
LRRK2: leucine-rich repeat kinase 2
MAP2: microtubule-associated protein 2
MOI: multiplicity of infection
MYTL1: myelin transcription factor 1-like
NBIA: neurodegeneration with brain iron accumulation
NCAM: neural cell adhesion molecule
ND: not determined
NEUROD1 or 2: neurogenic differentiation 1 or 2
NF200: high molecular weight neurofilament subunit
NGN2: neurogenin-2
NLS: nuclear localization signal
NT3: neurotrophin-3
NURR1: nuclear receptor related 1
OLIG2: oligodendrocyte transcription factor 2
PD: Parkinson's disease
PGK: phosphoglycerate kinase
PINK1: PTEN-induced putative kinase 1
PKAN: pantothenate kinase-associated
PNS: peripheral nervous system
PSA-NCAM: polysialylated-neural cell adhesion molecule
PSD95: postsynaptic density protein 95
PSEN 1 or 2: presenilin 1 or 2
PTB: polypyrimidine tract-binding protein
R: repsox
RA: retinoic acid
RanBP17: RAN binding protein 17
REST: RE1-Silencing Transcription factor
RT-qPCR: real-time quantitative reverse transcription PCR
S: SP600125
SB: SB202190
SMA: spinal muscular atrophy
SNP: single-nucleotide polymorphism
SOX2: SRY (sex determining region Y)-box 2
SYN: synapsin
SYT1: synaptotagmin-1
TGFβ: transforming growth factor beta
TH: tyrosine hydroxylase
TUJ1: neuron-specific class III beta-tubulin
Ub: ubiquitin
UNO: unoprostone
V or VPA: valproic acid
VACHT: vesicular acetylcholine transporter
vGLUT: vesicular glutamate transporter
VIM: vimentin
WB: western blot
Y: Y-27632.
